# Prophylaxis: Its Various Phases in Relation to Conservation of the Teeth1Read at the fifty-fifth annual session of the American Medical Association, in the Section on Stomatology, Atlantic City, June 7 to 10, 1904, copied from the Journal of the American Medical Association.

**Published:** 1905-02

**Authors:** Charles F. Allan

**Affiliations:** Newburgh, N. Y.


### PROPHYLAXIS: ITS VARIOUS PHASES IN RELATION TO CONSERVATION OF THE TEETH.[1]

BY CHARLES F. ALLAN, M.D., NEWBURGH, N. Y.

I WANT to make a broad distinction between reparative and restorative operations, and to weigh relatively the importance, from a tooth-saving point of view, of some prophylactic measures.

---

[1] Read at the fifty-fifth annual session of the American Medical Association, in the Section on Stomatology, Atlantic City, June 7 to 10, 1904, copied from the Journal of the American Medical Association.

Broadly speaking, prophylaxis, the prevention of disease, should be the highest aim of the physician. In our specialty of medicine we are nearer, perhaps, concrete facts than the general practitioner; the organs that furnish us our principal work are in sight and lesions are generally visible and within reach, and possibly it is not asking too much of the average patient to expect more of us in the way of prevention than in the warding off of disease by the family physician. Certainly if we do not accomplish quite as much, we are very culpable.

Prophylaxis is prevention—prevention of what? Not simply the loss of teeth by caries, nor added to it the loss of few or many teeth by pyorrhœa, but it means the prevention of the loss of the full comfortable use of thirty-two teeth through the whole or main part of possibly a long life. I do not think this at all a too high ideal to have before us. At present, in a large majority of cases, we certainly will not reach to this standard.

The great controlling factor in relation to immunity or non-immunity from caries has reference to the condition of the secretions of the mouth. We are now in the early stages of our examinations in the histo-chemistry of the saliva, and though we have a limited knowledge of the conditions making for decay, we are as yet unprepared and unable to change those conditions to one of immunity. Often we will not have the complete support of the patient in the matter of hygienic cleanliness, without which we are powerless to accomplish the best results; and it will also generally be impossible for any one person to have the consecutive management of many mouths extending over a series of years dating from childhood, without which the best services canot be rendered.

Still, prophylaxis is steadily gaining; hygienic cleanliness is being more and more insisted on by the dentist and more and more practised by our patients, and this greatly offsets environment. With better modes of treatment and a more careful living up to the high ideals of nature, we have every reason for encouragement.

The high standard of success here outlined in the salvation of the teeth involves of necessity a knowledge of the perfection of the normal conditions of the tissues and organs of the mouth; this in the environment of the teeth, in the teeth themselves and in their arrangement, and in the relation of the teeth of one jaw to the teeth of the opposing jaw.

I have placed at the head of these phases of necessary normal conditions that of environment; and yet if I am asked to describe what I consider a normal environment, mainly, of course, with reference to the juices of the mouth, I can only say I cannot tell. So far the chemical constitution of what may be called normal saliva has, to my knowledge, never been described and I think cannot be; that is, in its exhaustive and ultimate analysis within the varying limits of what still may be termed normal.

We occasionally hear of the rare case of an aged person who has never been to a dentist and who never has lost a tooth, and very, very much more rarely we meet such a person, but in what respect the saliva of this *rara avis* has differed from the mouth secretions of the ordinary individual we do not absolutely know.

We do know that Dr. Michaels (to whose investigations in the histo-chemistry of the saliva I will shortly refer) found that a hypoacid condition of the saliva was generally associated with rapid destruction of tooth tissue, and the hyperacid condition was associated with comparative immunity from decay. The researches of Drs. Kirk, Kyle, and others confirm those of Dr. Michaels. We know also that the immunity from caries in the rare cases I have referred to has not been as the result of extraordinary cleanliness. The deduction seems obvious.

The researches of Black, Williams, and others have proved to us that, in the main, the physical conditions of the teeth of different individuals of adult age are substantially the same, and the facts that are before us every day, showing the wide difference of conditions of tooth preservation, all point to a cause for tooth destruction, outside of the teeth themselves; this means, of course, in their environment, and this is now universally recognized.

In August, 1900, before the Third International Dental Congress, Dr. Michaels, of Paris, read a paper on "Sialo-Semeiology," which was a record, with deductions therefrom, of a long series of examinations and analyses in connection with the histo-chemistry of the oral secretions. The limits of my paper preclude even a *résumé* of this paper, but he clearly proved the intimate relation of immunity from caries with certain conditions found in the saliva, and he found radically different conditions of the saliva where caries in a mouth were rampant.

Though by no means exhaustive, Dr. Michaels's investigations were most carefully made, and his results and deductions have been amply confirmed by other investigators.

To quote a truism of medicine put in simple words, "It is a well-known clinical and laboratory fact that a study of the products of the secreting organs, which in their excreting functions throw off waste material, gives us by deduction a fair idea of what process is going on within the body." This being true, why, as an index of systemic condition, would not the study of the saliva give as good an idea as the study of the urine, especially as the latter is solely a waste product, while in the saliva, swallowed as soon as excreted, we have products of metabolism which return into the system as a part of a physiologic process?

The bearing of all this on tooth destruction is, of course, even more intimate than I have suggested above in connection with general systemic conditions, for the teeth are bathed in the secretions of the mouth and are exposed continuously to the fermentative and bacterial results of their environment.

The present relation of prophylaxis in this connection is as follows: We know the distinctive condition of the saliva, as found by Dr. Michaels and others, when related to rapid decay of the teeth; we know another distinctive condition of saliva seemingly always associated with immunity from decay; but I am afraid to say that as yet we know how to influence nutrition to that point that we can change the one undesirable condition to the one that means tooth preservation.

The study of the histo-chemistry of the saliva is one involving organic chemistry of the most intimate nature, and requiring an immense amount of laboratory detail and the study of formulæ and analyses requiring any amount of time. The advance already made is very great, and the introduction of the micropolariscope, according to Dr. Kirk, is a most valuable aid. To know the actual condition from which we require relief is a great point gained.

Drs. Kyle, Milligan, and others, in connection with hay-fever, ozæna, and diathetic and nervous diseases of various kinds, have gathered diagnostic indications from the saliva that have secured successful treatment and have proved to them of the greatest professional value. We are pointed in the right direction, with a solid substratum of facts to work up from. Laborers in this field are very necessary, and the reward, how to help secure immunity from caries, is the greatest that can be put before the dentist.

We cannot conceive of any period when absolute hygienic cleanliness in the mouth will not be necessary, but up to that time in the future when we shall be able to change by treatment the destructive character of the mouth secretions, this same hygienic cleanliness, as advocated and practised by its well-known exponent in Philadelphia, is, and will be, our best sheet-anchor for the preservation of the teeth. So much has been written by him, and his principles and practice are so well approved by the profession in the main, though not in all details, that it would be supererogatory for me to add any words.

Normal occlusion, that is, occlusion as opposed to malocclusion, as described so well by Dr. Angle in his record work on "Orthodontia," is a very necessary factor in prophylaxis. The knuckling up of the several teeth against one another, thereby securing for each tooth under stress the support of all the teeth of the arch, is most necessary and most helpful, and is provocative of comfort and tooth preservation. Now, when we add to this the perfect normal relation of the teeth of the mandible to the teeth of the upper jaw, that perfect relation of the planes and cusps of the teeth of the two jaws to one another, we have an ideal arrangement, a perfection of mechanical adaptation and artistic lines better than which we cannot conceive of; so, when we have before us a case of malocclusion to correct, it is this perfect ideal we must try to work up to, and we must always remember that the Almighty found thirty-two teeth none too many for the purposes of his perfect work, and it is not at all likely that we can do as well with less.

Now, the practical application of this matter of normal occlusion to prophylaxis lies in the interdependence of one tooth on another, as mentioned above. It lies in the fact that each tooth in a normal arch is necessary to the best welfare of every other tooth, and that you can not remove one tooth without injuring all. The natural tendency of the back teeth, in the absence of mesial support, is to move forward, and unfortunately that tendency is not to move *en masse,* but to follow the line of least resistance and seemingly pivot on the ends of the roots, the crowns tilting, with the effect of not only destroying to a great extent the mutual support of adjoining teeth, but also destroying more or less that perfect relation of the teeth of the two jaws to one another.

All irregularities of the teeth, and especially all losses of teeth, are provocative of further trouble. The proper interdigitation of the cusps of the teeth of one jaw in the sulci of the teeth of the opposing jaw is interfered with, the area of masticating surface is reduced, and what remains is usually not in as good condition for service. Some of the teeth no longer have the stress of use along the line of the axis of the roots, and the tilting continuously increases. The malocclusion affects also certain teeth that otherwise would be in normal health by putting on them unusual strain, and that strain in a direction the teeth are not expected to bear, with the result of loosened teeth in diseased sockets. The tendency of all such maloccluded teeth is to get lame and loose and to be early subjects for pyorrhœa, and in the end the forceps. Teeth irregularly placed in the jaws are difficult to keep clean, and are much more prone to caries, and in more ways than I have time to speak of malocclusion tends to tooth loss. Proper occlusion, especially when attended to early in life, is an eminently important factor in prophylaxis.

Tooth loss is mainly occasioned by departure from the high ideals established for us by Nature. The teeth, generally erupting with normal shapes and with normal structure and physical conditions, are, by reason of perverted environment, subjected to caries. Caries generally means repair, and, in the fullest sense of the word, should mean restoration; and it is this in many cases incomplete repair and hardly at all restoration that is a great cause of tooth destruction. To give a broad generalization in one short clause, we should live up to nature.

All of these many years, in which we have made so many and great advances as a profession, we have been handicapped by the fact that we have had no filling-material that was at all ideal. Amalgam, tin, gutta-percha, cements, have all done us great and good service, and, above all, gold. The latter is a royal metal, and has done our patients a royal amount of good, but restoration has been a restoration of contour only; not a restoration of color and appearance; not a restoration of thermal non-conductivity; not a restoration of the vitreous polished enamel surface which is of such immense importance; and this lack of restoration has generally been at the expense of serious distress to our patients; often of serious physical and nervous strain to the operator. Drs. Varney and Webb, and others as well, practically gave their lives to establishing their high ideals of tooth restoration, and then their ideals failed in the above-mentioned serious respects. All glory to them, however; they were in advance of their fellows, and we are greatly better dentists by having had their ideals to work up to.

Now the relation of all this to prophylaxis lies in the fact that in just these important matters in which tooth restoration fails with gold, it is a success with porcelain. The vitreous polished surface, practically continuous with the enamel surface, the non-conductivity of the material, its comparative ease of insertion at an immense saving of time, pain, and nervous strain, are all important respects in which the prophylactic value of porcelain transcends that of other materials.

Again, I cannot go into details, but it is entirely practical in the great majority of cases to fill the occluso-proximal cavities of the bicuspid and molar teeth with porcelain, at a great saving of strain and pain to the patient, with a perfect restoration of contour made in the furnace and not in the patient's mouth. Some of you, I fear, will dissent from this statement, but I want to go on record as saying that it is practical, and that such restorations, if properly made, will be permanent.

For the sake of brevity, I have instanced only occluso-proximal cavities, because they are, possibly, the most difficult and the necessities of such cases are greater, but the principles involved are the same with all proximal fillings, and the essential saving conditions are just as evident.

The last few years have developed an immense amount of literature on prophylaxis, but it has been of the narrow kind that has seen but one phase of the subject, and for that has claimed the earth. All of you know that prophylaxis is many-sided,—has, indeed, many more sides than I have had time to indicate,—and that though hygienic cleanliness is one phase, and that a very important one, it is still not all there is that stands for the prevention of the loss of the teeth.

The investigations of Michaels, which include an immense amount of painstaking laboratory work, have opened up a field of activity whose promise is very great.

In the language of the very reserved and modest claim made by the eminent editor of the *Dental Cosmos,* "Dr. Michaels has thrown a ray of scientific light on the problem of dental prophylaxis, and has clearly indicated the direction from which greater light on the problem may be expected to come."

Dr. Angle, in his great work on malocclusion, has treated his subject in a scientific manner quite beyond anything hitherto attempted, and in the simplicity of his classification and in the clear idea he has given us of the ideal we are always to work up to, with methods and apparatus most simple of use, has given the profession great aid in treatment, which in so many cases makes for prophylaxis.

To this should be added a word of tribute to the enthusiastic and earnest labors of Dr. Jenkins, stretching over many years and all to the end that tooth-structure might be imitated and that tooth repair might mean tooth restoration in the fullest and best sense of the word.

Other names could be added to this brief list, but these I have mentioned seem to me typical of the best, in that they have obtained their important results only because of great labor, backed up by most earnest enthusiasm.

It is such enthusiasm that in the end will conquer for tooth salvation. It is such enthusiasm that always conquers.

